# Genome-wide transcriptional profiling for elucidating the effects of brassinosteroids on *Glycine max* during early vegetative development

**DOI:** 10.1038/s41598-019-52599-3

**Published:** 2019-11-06

**Authors:** Li Song, Wei Chen, Qiuming Yao, Binhui Guo, Babu Valliyodan, Zhiyong Wang, Henry T. Nguyen

**Affiliations:** 1 Joint International Research Laboratory of Agriculture and Agri-Product Safety, Jiangsu Key Laboratory of Crop Genomics and Molecular Breeding, Yangzhou University, Yangzhou, 225009 China; 2 Division of Plant Sciences, University of Missouri, Columbia, MO 65211 USA; 3 Department of Computer Science, Christopher S. Bond Life Sciences Center, University of Missouri, Columbia, MO 65211 USA; 4 Department of Plant Biology, Carnegie Institution for Science, Stanford, CA 94305 USA

**Keywords:** Plant sciences, Brassinosteroid

## Abstract

Soybean is a widely grown grain legume and one of the most important economic crop species. Brassinosteroids play a crucial role in plant vegetative growth and reproductive development. However, it remains unclear how BRs regulate the developmental processes in soybean, and the molecular mechanism underlying soybean early development is largely unexplored. In this study, we first characterized how soybean early vegetative growth was specifically regulated by the BR biosynthesis inhibitor propiconazole; this characterization included shortened root and shoot lengths, reduced leaf area, and decreased chlorophyll content. In addition, the growth inhibition induced by Pcz could be rescued by exogenous brassinolide application. The RNA-seq technique was employed to investigate the BR regulatory networks during soybean early vegetative development. Identification and analysis of differentially expressed genes indicated that BRs orchestrate a wide range of cellular activities and biological processes in soybean under various BR concentrations. The regulatory networks between BRs and multiple hormones or stress-related pathways were investigated. The results provide a comprehensive view of the physiological functions of BRs and new insights into the molecular mechanisms at the transcriptional level of BR regulation of soybean early development.

## Introduction

Hormones function as important regulators of growth and development in both plants and animals^1,2^. Brassinosteroids (BRs), which are plant-specific steroid hormones, have a wide range of effects on plant growth and development processes, including cell elongation and division, plant architecture, biomass, photomorphogenesis, root development, and seed germination^3–7^. Moreover, BRs also mediate plant responses to various abiotic and biotic stresses, such as heat, salt, drought, cold, oxidative and heavy metal stresses; pathogen attack; and herbicide/pesticide tolerance^8–10^. Although the importance of BRs has been recognized in *Arabidopsis*, our understanding of BR function remains incomplete in crop species. The main reason is that few null mutations have been reported in crop species^11,12^, which leading to the direct regulation of target genes by BR signaling that is difficult to identify.

Soybean is a grain legume that is grown worldwide and provides protein and oil for humans. Several physiological studies have revealed that BRs are involved in the growth and development of soybean. BRs promote soybean epicotyl elongation by inducing a functional xyloglucan endotransglycosylase that is highly expressed in inner epicotyl tissue during elongation^13,14^. Foliar applications of BRs prior to water-deficit stress treatments could partially alleviate the detrimental stress effects on the growth of soybean by promoting the accumulation of osmoprotectants and dry weight and by causing decreased accumulation of malondialdehyde (MDA) and decreased electrical conductivity^15^. In addition, the physiological effects of BRs on soybean are also correlated with different genotypes and concentrations of BR. For example, BRs differentially regulate nodule formation based on soybean genotype^16^. After soybean plants were incubated with 0.1 µM to 10 µM of epi-brassinolide, their total nodulation, plant wet weight, root length, shoot length, first internode length, and number of lateral roots were reduced within 3 weeks^17^.

Subsequent genetic studies indicated that BR signal transduction and synthesis-related genes corresponded to those physiological phenotypes. For example, GmCPDs catalyzing BR synthesis have been shown to be involved in the regulation of early stages of flowering^18^. Transgenic plants overexpressing *GmBZL2*^*P216L*^ or *GmBZL3*^*P219L*^ (*AtBZR1*-like gene) could partially rescue the defects of *bri1-5 Arabidopsis* mutants, which suggests that GmBZL functions are conserved between soybean and its homolog in *Arabidopsis*^19,20^. Similarly, soybean cultivars with relatively large pods are inclined to have a relatively high *GmBZR1* expression level in the pods^21^. Furthermore, the growth defects of the BR-insensitive mutant *bri1-5* can be complemented by overexpressing *GmBRI1*^22,23^.

These extensive physiological and genetic findings indicate that BRs play important roles during soybean development and that the identified major signaling components in soybean coordinate soybean development. However, the biological functions of BRs have not been fully investigated, and the detailed regulatory mechanisms of BRs are not well characterized in soybean. In recent years, the discovery and use of BR-specific biosynthesis inhibitors have provided an alternative way to determine the physiological functions of BRs in crop species^24–26^. Among these inhibitors, propiconazole (Pcz) has been reported to be a specific and affordable BR biosynthesis inhibitor for *Arabidopsis* and maize. Pcz treatment produces typical BR-deficient phenotypes of *Arabidopsis*, such as short primary roots, dark-green cotyledons and reduced hypocotyl length^25^. In addition, large-scale RNA-seq transcriptome analyses have also revealed BR-regulated gene expression networks in *Arabidopsis*, *Gerbera hybrid*, etc.^27–29^.

Exploring how BRs regulate soybean development by identifying BR-regulated gene expression will help to bridge this gap. It is also very helpful to understand how BR participates in responses to biotic and abiotic stresses in soybean. Thus, the main objective of this study was to identify genes in the soybean early vegetative growth stage that are differentially expressed in the presence of a BR synthesis inhibitor (Pcz) with or without exogenous brassinolide (BL) application by the RNA-seq method. In addition, the physiological effects and the related biological pathways in response to BRs were studied to determine possible connections between gene expression levels and physiology. Our results demonstrated that Pcz is a specific BR biosynthesis inhibitor for soybean and that BRs play important roles in soybean growth and development. The biological significance of the soybean transcriptomic response under various BR concentrations is discussed.

## Results

### Brassinosteroids modulate soybean early vegetative growth *in vivo*

**Figure 1:**
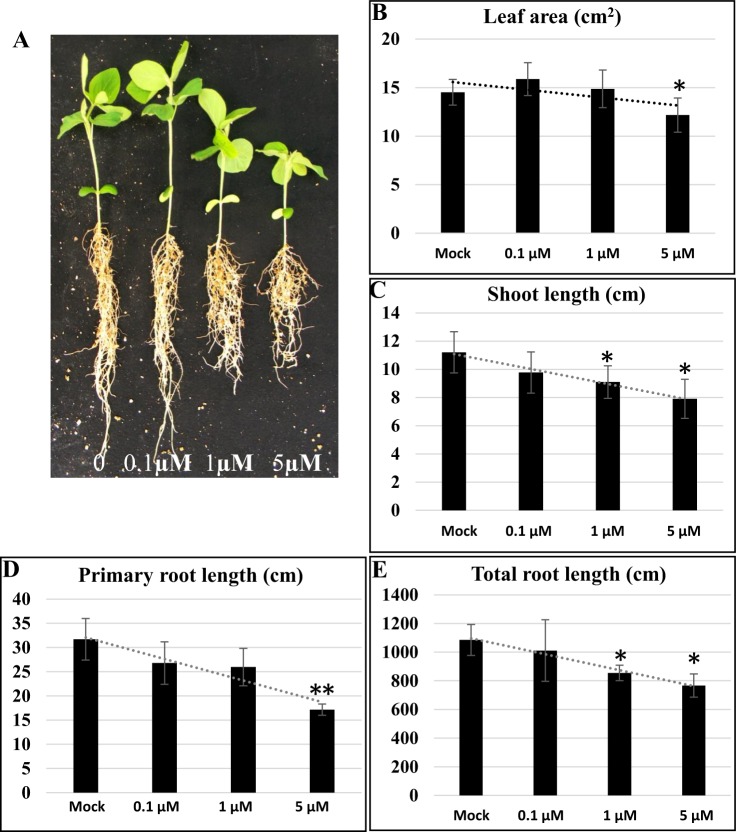
Responses of soybean seedlings to treatments with different concentrations of the inhibitor Pcz. (**A**) Left to right: soybean (Williams 82 genotype) growing until the VE stage and then irrigated with water with different concentrations of Pcz for 10 days. (**B**–**E**) Leaf area, shoot length, primary root length and total root length were monitored under 0.1 µM, 1 µM or 5 µM Pcz treatment for 10 days after soybean growth reached the VE stage. (n ≥ 30). Statistical differences are marked with *(p ≤ 0.05) or **(p ≤ 0.01) based on Student’s *t*-test analysis.

To investigate whether and how BR biosynthesis affects soybean growth, the morphological phenotypes of Williams 82 soybean seedlings treated with three different concentrations of Pcz for 10 days were evaluated. As shown in Fig. 1A, the treatments resulted in dose-dependent reductions during soybean seedling growth, where 5 µM Pcz significantly decreased the elongation of the shoots and the total lateral root length by 30%, the primary root length by 45%, and the leaf area by 16%, although the leaf area of soybean increased slightly under low concentrations (0.1 µM Pcz) (Fig. 1B–E). Previous study has reported that light-grown BR-deficient *det2* mutant shows a dark-green phenotype due to the effects on the light-regulated seedling development^30^. Here, we found that the leaf chlorophyll content increased with increasing concentrations of Pcz applied (Fig. S1A,B). In addition, the growth defects, including those in plant height, shoot length, petiole length and leaf area, could be partially rescued by adding exogenous BL at different concentrations (Fig. S1C–F). These results suggest that BR synthesis specifically regulates soybean early vegetative growth and that this growth inhibition induced by Pcz could be compromised by exogenous BL applications.

### Identification of differentially expressed genes under various BR concentrations

**Figure 2:**
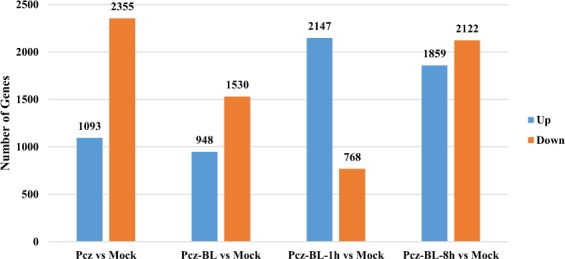
Number of DEGs between mock and various treatments. Number of DEGs in Williams 82 soybean plants under the following conditions (Pcz vs Mock): 5 µM Pcz for 10 days, (Pcz-BL vs Mock): 5 µM Pcz with 10 nM BL for 10 days, (Pcz-BL-1h vs Mock): 5 µM Pcz for 10 days followed by 1 µM BL for 1 h, (Pcz-BL-8h vs Mock): 5 µM Pcz for 10 days followed by 1 µM BL for 8 h] (P value ≤ 5e-5, and genes with an expression ratio log2 ≥2 or ≤−2 were selected).

Transcriptional regulation during BR signal transduction plays an important role in plant development. BZR1 and BZR2/BES1 have been reported to directly regulate the expression of downstream target genes in the BR signaling pathway^31,32^. To further explore the influence of different BR levels on soybean growth and define the impact of BRs in a broader context, genome-wide gene expression research was performed in soybean at the V1 stage (first-node: fully developed leaves at the unifoliate node). Soybean plants irrigated with water (10 days) were used as mock controls. The treatments were performed as follows: the plants were incubated with either 5 µM Pcz or 10 nM BL for 10 days (indicated as Pcz-BL) or incubated only with 5 µM Pcz for 10 days (indicated as Pcz), or the Pcz treatment was complemented with 1 µM BL exogenously applied for 1 h or 8 h (indicated as Pcz-BL-1h and Pcz-BL-8h, respectively). As shown in Fig. 2, 2,478 to 3,981 genes showed significant changes (P ≤ 5e-5, fold change ≥2 or ≤−2) under the different treatments. In total, 9,084 genes were found to be differentially regulated in soybean seedlings under at least one treatment: Pcz, 3,448 genes; Pcz-BL, 2,478 genes; Pcz-BL-1h, 2,915 genes; and Pcz-BL-8h, 3,981 genes. Among them, the Pcz-BL-8h treatment induced the largest number of significant changes in transcript abundance: 2,122 downregulated and 1,859 upregulated genes (Fig. 2).

### Brassinosteroids regulate a wide range of cellular activities and biological processes in soybean

**Figure 3:**
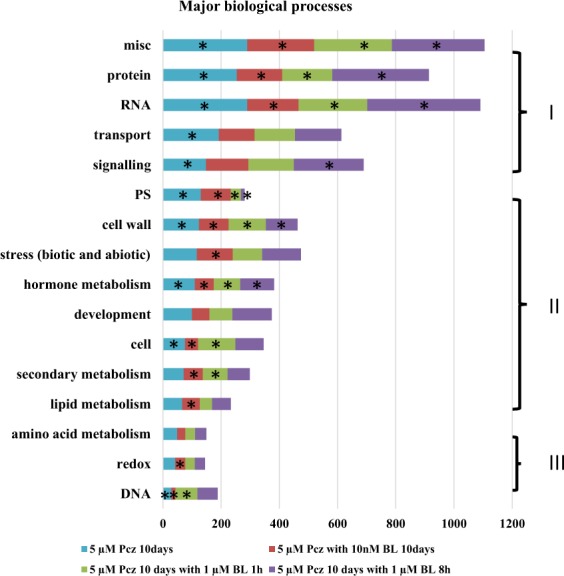
GO term assignments in different categories of biological processes for DEGs by Mapman tool. The abscissa indicates the number of unigenes. The same color represents the same conditions. The graph illustrates the number of genes annotated with 16 different GO terms. Four groups (groups I, II, III and VI) were divided according to the number of genes in response to different conditions: group I > 600 genes, 200 genes < group II < 600 genes, and 100 genes < group III < 200 genes. The statistical significance of each process under all treatments was assessed by Fisher’s exact test. The overrepresented categories of biological processes (P values < 0.05) were labeled with an asterisk (*).

To understand what biological processes were altered in soybean under different BR levels, the identified DEGs were further functionally classified by using MapMan bin code^33^. Compared with the results of annotated biological processes, approximately 62–66% of DEGs fell into different pathways. Four functional categories (groups I, II, and III: major biological process; group IV: minor biological process) were divided based on the number of responsive genes (Fig. 3 and S2). The total number of responsive genes for group I (largest group) was more than 600, including miscellaneous (1,105), protein (914), RNA (1,091), transport (613), and signaling (690) functional categories. Regarding protein metabolism categories, the DEGs were significantly associated with posttranslational modification and degradation pathways. Regarding RNA categories, the DEGs were rich in transcription factor families, including *bHLH*, *AUX*/*IAA*, *ARF*, *AP2*/*EREBP*, *MYB* and homeobox genes under each treatment. In addition, the biological processes related to miscellaneous, protein and RNA were overrepresented under all treatments in group I.

The total number of responsive genes for group II was between 200 and 600, which included the photosynthesis (PS) (281), cell wall (463), stress (474), hormone (382), and development (374) functional categories. Among these categories, the most highly regulated pathways involved PS, the cell wall and hormone metabolism processes. Group III corresponded to amino acid metabolism, redox, and DNA processing processes. In group IV, the total number of responsive genes was fewer than 100 (Fig. S2). Most of the group IV processes were involved in energy production. Compared with those in groups I and II, a few processes in groups III and IV significantly changed under all treatments.

### Overview of different treatments resulted in impacts on the soybean transcriptome

To explore the variation tendency of DEGs among the different treatments, the DEGs in group I and group II were studied. As shown in Fig. S3 (group I), most of the DEGs under the Pcz or Pcz-BL treatments were downregulated. However, more DEGs were upregulated under Pcz-BL-1h or Pcz-BL-8h treatments than under the Pcz or Pcz-BL ones. Similar regulatory trends were also found in group II (Fig. S4). Interestingly, cell-related biological processes were mostly inhibited in the Pcz-BL-1h treatment (Fig. S4). In addition, although the total number of stress (biotic and abiotic)-related DEGs was similar under the different treatments, their regulatory tendencies were the opposite. These results not only indicated that the Pcz and Pcz-BL treatments resulted in a major inhibitory impact on the soybean transcriptome but also indicated the integration between transcriptional changes and physiological acclimation.

**Figure 4:**
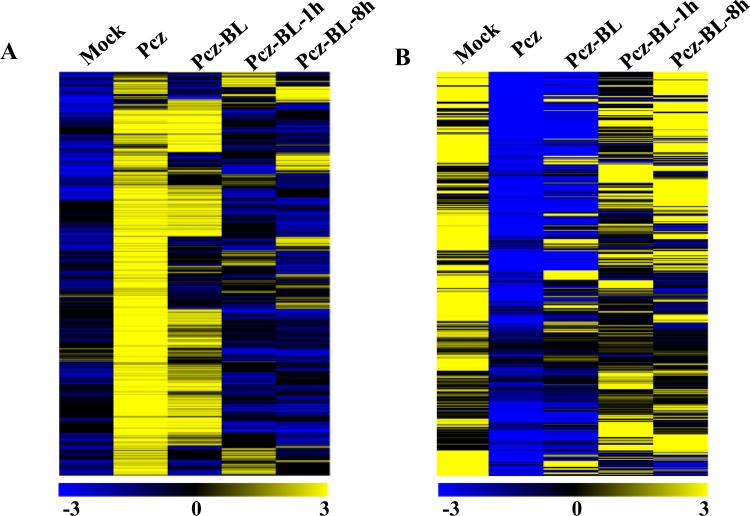
Heat map representation of the FPKM expression of upregulated or downregulated DEGs in Pcz treatment under various BR concentrations. The color bar at the bottom shows the range of expression values, from increased expression (yellow) to decreased expression (blue).Genes were hierarchically clustered based on Euclidean distance of FPKM data and complete linkage. (**A**) Heat map for upregulated DEGs between Pcz treatment and mock (**B**) Heat map for downregulated DEGs between Pcz treatment and mock.

**Figure 5:**
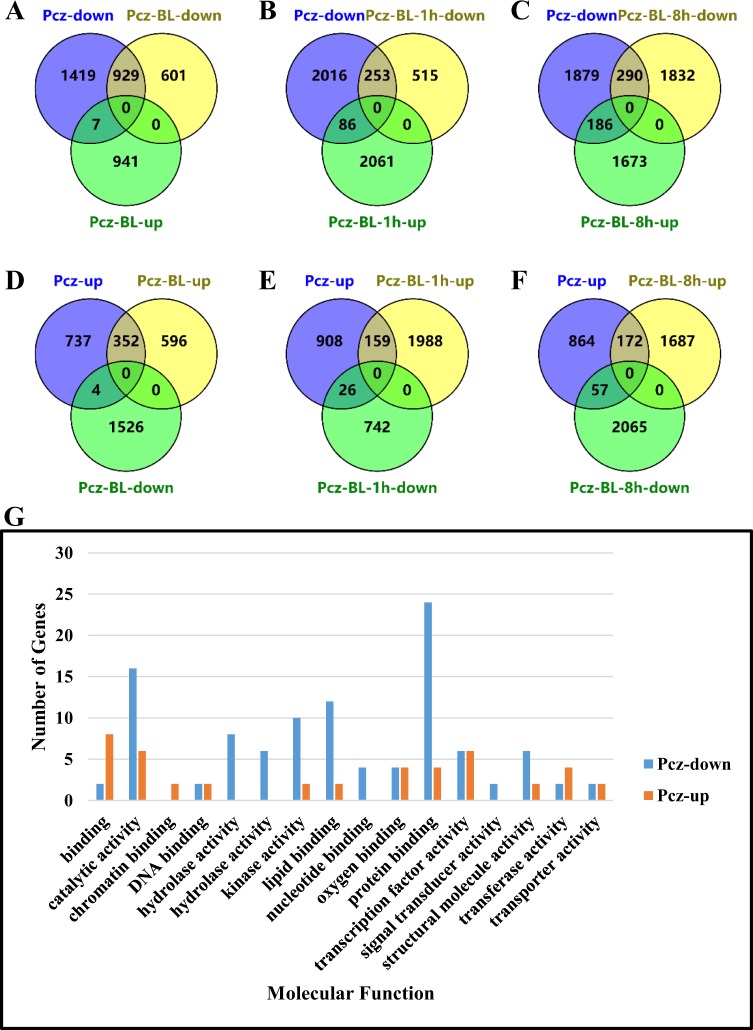
Venn diagram and GO terms display the upregulated or downregulated overlapping DEGs between Pcz treatment and other treatments (Pcz-BL, Pcz-BL-1h and Pcz-BL-1h). (**A**–**C**) Venn diagram showing the overlapping DEGs between the downregulated genes under Pcz treatment and other treatments (Pcz-BL, Pcz-BL-1h or Pcz-BL-1h). (**D**–**F**) Venn diagram showing the overlapping DEGs between the upregulated genes under Pcz treatment and other treatments (Pcz-BL, Pcz-BL-1h or Pcz-BL-1h). (**G**) GO terms display the inversely regulated DEGs between Pcz treatment and other treatments (Pcz-BL, Pcz-BL-1h or Pcz-BL-1h) as molecular function. The absolute number reflects the amount of GO IDs that were connected to the molecular function.

The distributions of DEGs that were commonly upregulated or downregulated under the Pcz treatments are those likely affected by BR directly and indirectly. Comparison of the expression dynamics of those DEGs under the other three treatments will help to elucidate how soybean response to BL treatment at an early or late stage. As shown in Fig. 4, the expression levels of the DEGs under Pcz treatment were recovered or partially recovered under Pcz-BL-1h or Pcz-BL-8h treatments. Conversely, a similar distribution between Pcz and Pcz-BL treatments was observed. The overlapping of DEGs between the downregulated or upregulated genes under Pcz treatment and other treatments was further illustrated in a Venn diagram (Fig. 5). Out of 2355 downregulated DEGs under Pcz treatment, 929 genes have shown reduced expression pattern under Pcz-BL treatment, but only 253–290 genes showed reduced expression pattern under Pcz-BL-1h or Pcz-BL-8h treatments (Fig. 5A–C). Similarly, there are less overlapping genes between Pcz and Pcz-BL-1h or Pcz-BL-8h treatments compared with Pcz-BL treatment (Fig. 5D–F). A closer look at the distribution of molecular function with those reversely regulated DEGs showed that the GO terms for protein binding, catalytic activity, and transcription factor activity were more abundant (Fig. 5G). Overall 313 reversely regulated DEGs were identified (Tables S1 and S2). Cell wall pathway-related transcripts were enriched and reduced early under Pcz treatment, but highly increased after BL applied after 8 h (Table S1). However, the genes related to amino acid metabolism, cell organization and division responded quickly and changed their expression pattern differently after BL applied after 8 h (Table S2). These results indicated that the majority of DEGs caused by depletion of BR is actually from BR deficiency and the expression level of DEGs is relevant to the time point of BR application and that short-term (several hours) applications of exogenous BL could dramatically alter gene expression patterns.

### Overview of the primary and secondary metabolism pathways under various BR concentrations

**Figure 6:**
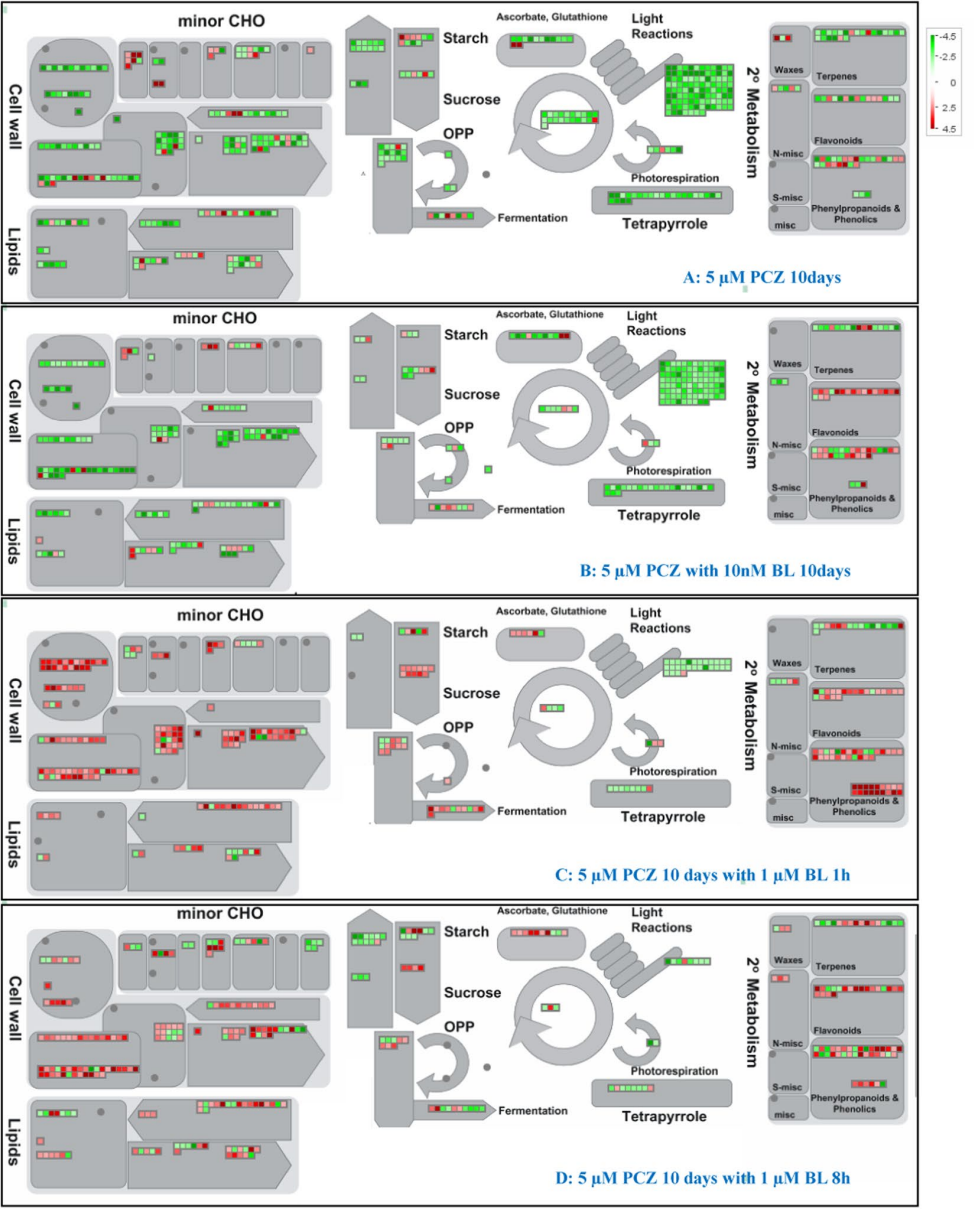
MapMan overview of the metabolism- and cell wall-related genes among group I that were differentially expressed under the four different treatments. Metabolism-, PS-, and lipid-related genes were downregulated under Pcz or Pcz-BR treatment but upregulated after high concentrations of BL were applied for 1 h or 8 h compared with mock treatment. (**A**) 5 µM Pcz for 10 days, (**B**) 5 µM Pcz with 10 nM BL for 10 days, (**C**) 5 µM Pcz for 10 days with 1 µM BL for 1 h, (**D**) 5 µM Pcz for 10 days with 1 µM BL for 8 h. The log2-fold changes of significantly DEGs were imported and visualized in MapMan. Transcripts significantly up- and downregulated are indicated in red and green, respectively. The genes were assigned to their associated metabolic pathways. Each square corresponds to a gene. The green squares indicate genes suppressed in comparison to the mock. The red squares indicate genes induced in comparison to the mock. MapMan version 3.6.0 was used to generate the images from the gene expression data. The scale bar is shown in log2 from −4.5 to 4.5.

To gain a better understanding of transcriptional shifts under different treatments in soybean, an overview of the primary and secondary metabolism pathways in group I was generated by using MapMan hierarchical ontology software (Fig. 6). The analysis indicated downregulation of organellar energy metabolism and secondary metabolic process-related genes under the Pcz and Pcz-BL treatments, whereas upregulation occurred under the Pcz-BL-1h and Pcz-BL-8h treatments. In these classes, 116 light reaction-related transcripts were downregulated under the various treatments, especially under the Pcz and Pcz-BL treatments. Transcripts in these categories include LHC (35) (photosynthetic pigment-related genes), PSB and PSA (61) (components of photosystem I or II), ATP synthesis (4), and electron carriers (16). The levels of both transcripts encoding several cell wall-involved components and transcripts involved in minor carbohydrate metabolism were perturbed in response to the various treatments, which indicated that cell wall modification and degradation may accelerate during the response to changes in BR levels. Specifically, transcripts involved in plastid/light reactions were significantly altered, which further indicated that BRs have cell-wide impacts on central energy metabolism (Fig. 6). All of the results demonstrated that BR synthesis plays a vital role in soybean development. Inhibition of BR biosynthesis imposes negative effects on metabolic processes in soybean, which may consequently slow soybean seedling growth.

### Multiple hormone biosynthesis and signal transduction pathways are reprogrammed under various BR concentrations

Different hormone signal transduction and synthesis-metabolism interactions play important roles during plant development^34^. To elucidate the hormone networks associated with BRs in soybean, the expression patterns of hormone-related transcripts were investigated in this study. Interestingly, we found that auxin, abscisic acid (ABA), gibberellin (GA), ethylene, jasmonates (JAs), and cytokinins (CKs) were involved in BR-response networks in soybean. Among them, ethylene response-related genes (91 genes) correspond to the largest group (Table S3). Three kinds of ethylene receptors, *ETR2* (ETHYLENE RECEPTOR2), *EIN4* (ETHYLENE-INSENSITIVE4) and *ERF9* (ETHYLENE RESPONSE FACTOR9), were found to be regulated. Many genes encoding members of the 2-oxoglutarate Fe(II)-dependent oxygenase superfamily, which are involved in the ethylene metabolism process, have also been found to be regulated under various BR concentrations. Second, auxin synthesis and signaling pathway-related genes correspond to the second largest group (Table S3). Several auxin transporter-related genes (*AUX1*, *PIN1*, *PIN3*, *PIN5* and *PIN7*) and synthesis-related genes (*GH3.1*, *GH3.6*, *GH3.9*, and *GH3.10*) were downregulated under BR-deficient conditions. Further, several auxin synthesis-related genes (*GH3.1*, Glyma.02G125600; *GH3.6*, Glyma.06G260800) were upregulated under Pcz-BL-8h treatments. GA20-oxidase and GA2-oxidase control GA biosynthesis and degradation, respectively, and are responsible for regulating local GA levels in the SAM and leaf primordia^35–37^. In our study, we found that GA20-oxidase (Glyma.06G155000) is highly induced by Pcz-BL-1h and Pcz-BL-8h (Table S3). Therefore, BR and GA may act interdependently via a direct link between BZR1 and GA synthesis or degradation.

Genes involved in the pathways of other hormones were also identified: ABA (23 genes), GA (22 genes), JAs (30 genes), SA (3 genes) and CKs (18 genes) (Table S3). Among those genes, several JA pathway-related genes were found in our RNA-seq experiments, including *LOX1*, *LOX2*, *LOX3*, *LOX5*, *AOC3*, *AOC4*, and *SMT*. ABA biosynthesis and response-related genes, including *NCED1*, *NCED4*, *AREB3*, *ABA1*, and *ABA3*, showed different expression patterns. A few CK-related genes such as *ACO1*, *ACO4*, *ACS6*, and *ACS10* were found to be regulated under various BR concentrations. In addition, we found that *BES1* (Glyma.06G034000), a well-characterized transcription factor involved in the BR signaling pathway, was downregulated under Pcz-BL condition. *DET2* (Glyma.11G110300), which encodes a steroid 5α-reductase in the BR biosynthesis pathway, was upregulated under Pcz treatment. Taken together, these results indicated not only that soybean BR signaling pathways may play a central role in coordinating growth and development by interacting with other hormones such as auxin, GA, ethylene, JAs, and CKs but also that the synthesis and signal transduction of BRs are strictly regulated by a feedback mechanism in soybean.

### Transcriptional regulatory network analysis revealed BR biosynthesis involvement in biotic and abiotic stress-related pathways

Previous studies have shown that BRs play a critical role in plant disease and immune responses^38^. However, in contrast to the knowledge of BR-mediated defense responses in *Arabidopsis*, barley, rice and tobacco, little information is available on soybean^39–41^. In this study, 152 biotic stress-related genes exhibited altered expression patterns in soybean under varying levels of BRs (Table S4). Among them, 50 transcripts associated with biotic/respiratory burst/signaling pathways were found. Enhanced disease susceptibility1 (EDS1) and PAD4 are well-known regulators that mediate plant defense^42^. It was reported that two soybean EDS1 isoforms are required for bacterial resistance to *Pseudomonas syringae* pv. *glycinea 2* (*Rpg2*)^43^. Here, we found that two genes encoding GmEDS1 (Glyma.06G187300) and GmPAD4 (Glyma.08G002100) were upregulated after exogenous BL application. R proteins mediate the interactions between plants and biotrophic bacteria and regulate resistance to bacterial invasion^44^. Notably, as the most common type, NBS-LRR type R proteins have been reported to play an important role in soybean resistance to SCN and to strains of *Pseudomonas syringae* pv. *glycinea* or in restricting nodulation in soybean^45–47^. In this study, a large number of R genes were identified as being induced by Pcz-BL treatment (Table S4); these genes accounted for more than 67% of the total number of R genes. Among them, the number of encoded R proteins containing a nucleotide-binding site and leucine-rich repeat (NBS-LRR) domain exceeded 1/3 of the total. Our results support that the changes in BR contents play a vital role in mediating the soybean defense response by regulating the expression patterns of R genes, especially NBS-LRR type R genes.

BRs provide tolerance against heat, cold, drought, and salt stress by controlling many stress responses that help plants adapt to adverse conditions^48^. Here, 134 abiotic stress-related genes were identified in response to various BR concentrations (Table S4). The largest number of genes found were heat and drought/salt related (84 genes), which accounted for more than half of the abiotic stress-related genes. Among them, heat shock proteins (HSP70, HSP17, HSP81, HSP15, HSP26, HSP22, HSP31, etc.) and dehydration-responsive proteins (ERD15, ERD4, and ERD22) accounted for the majority. In addition, a few cold- and wound/touch-related genes were found to be regulated under various BR concentrations. These findings indicate that BRs function to regulate many abiotic stress-response genes, especially those involved in dehydration and the heat response, in soybean.

### BRs participate in soybean root development under water-deficit conditions with synergistic or antagonistic effects

Several studies have revealed that BR-deficient or BR-insensitive mutants have enhanced tolerance to drought^49–51^. Recently, transcription factor RESPONSIVE TO DESICCATION 26 (RD26) was shown to mediate the crosstalk between drought and brassinosteroid signaling pathways^52^. It was reported that the transcripts associated with several plant hormone synthesis and signaling pathways were regulated under water-deficit conditions in soybean roots, although few BR-related genes have been found^53^. To elucidate the regulatory relationships between BRs and water-deficit stress, the expression patterns of hormone- and stress-related genes regulated by Pcz or BL in this study were extracted from datasets of soybean roots under various water-deficit conditions (Table S3, S4).

First, many hormone-related genes that respond to BRs are involved in the water-deficit response of soybean roots (Table S3). For example, many homologs of members of the 2-oxoglutarate Fe (II)-dependent oxygenase superfamily were regulated under water-deficit conditions. The expression level of *ABA1* (Glyma.11G055700) was upregulated under mild stress (MS) and severe stress (SS) conditions but downregulated under Pcz-BL-8h. The expression levels of *DWF1* were downregulated under various water-deficit conditions. *LOX1* (Glyma.08G189600) are JA biosynthesis pathway-related genes, was increased under Pcz treatment, but were decreased under Pcz-BL-1h treatment and MS or SS conditions. The expression levels of ACC oxidase (related to the CK pathway, Glyma.06G117200) increased under Pcz-BL-1h and Pcz-BL-8h treatment, but decreased under Pcz and Pcz-BL treatment, including SS and SR conditions. These results indicated that BRs play an important regulatory role between hormone and water-deficit interactions.

Second, many stress-related genes that respond to BRs are also involved in the water-deficit response (Table S4). For example, several ATRBOHBs, which encode respiratory burst oxidase homolog B, were upregulated under Pcz and Pcz-BL. In addition, two of them (Glyma.03G236300 and Glyma.10G152200) were upregulated under MS condition. One pathogenesis-related thaumatin family gene (Glyma.06G023900) was downregulated under Pcz-BL and Pcz-BL-1h treatments, and it could be highly reduced under MS, SS and SR conditions. *RD22* (Glyma.13G283900), a dehydration-responsive gene, was repressed under Pcz-BL treatment and induced under Pcz-BL-1h, Pcz-BL-8h treatment and MS and SS conditions. One *HSP17.6* gene (Glyma.06G054700) was upregulated under Pcz and Pcz-BL treatments, and induced under MS and SS conditions also. All of the above results indicated that numerous BR-responsive genes also participate in the response to water-deficit stress, which suggests that BRs might synergistically or antagonistically affect the water-deficit stress response of soybean roots.

### Validation of RNA-seq data

**Figure 7:**
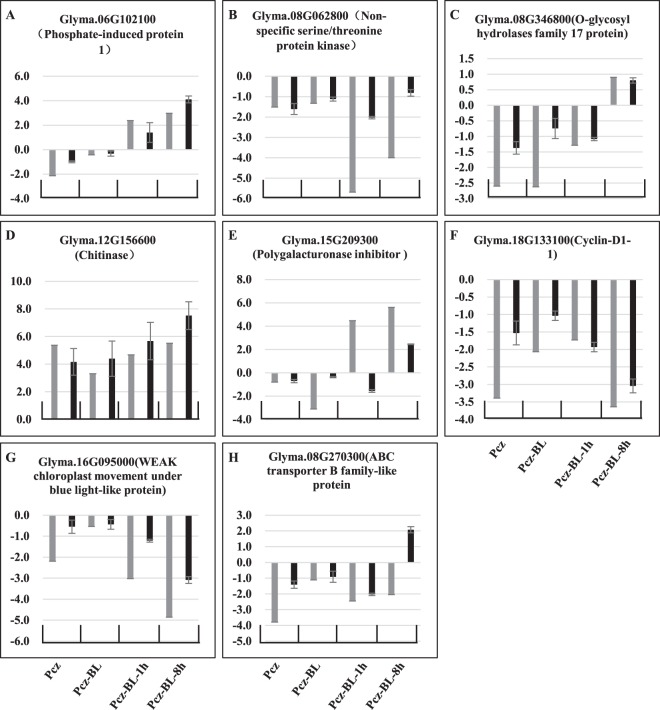
Validation and comparison of eight differently expressed genes selected from RNA-seq analysis by qRT-PCR. qRT-PCR data were normalized to the stable endogenous control (Cyclin gene). The fold changes are presented as mean with standard errors (SE) of three biological replications. RNA-seq results showed in gray and qRT-PCR results showed in black.

Eight genes were randomly selected from the DEG list for qRT-PCR analysis to validate the RNA-seq expression data and its reliability. As shown in Fig. 7, three genes (Glyma.08G062800, Glyma.18G133100 and Glyma.16G095000) showed a downregulation pattern under all treatments (Fig. 6B,F,G). However, the transcript of Glyma.12G156600 was significantly induced under all conditions (Fig. 7D). Except for the pattern of one gene (Glyma.06G102100), which showed a slightly increased expression under the Pcz-BL-1h treatment, the expression patterns of the other four genes were slightly reduced under the Pcz, Pcz-BL, and Pcz-BL-1h treatments but increased under the Pcz-BL-8h condition (Fig. 7A,C,E,H).

The expression patterns of most genes were similar between the qPCR and RNA-seq data. However, Glyma.15G209300 and Glyma.08G270300 showed opposite expression patterns under Pcz-BL-1h and Pcz-BL-8h, respectively. Therefore, correlations between the RNA-seq and qRT-PCR data were analyzed using log2 expression levels. As shown in Fig. S5, the qRT-PCR measurements were highly correlated with the RNA-seq results (R^2^ = 0.7895). Thus, the expression patterns of these genes from the qRT-PCR results were highly consistent with the results from the RNA-seq, which indicated the fidelity and reproducibility of the RNA-seq analysis.

### Comparison of BR-induced or repressed genes between *Arabidopsis* and soybean

Microarray and RNA-seq studies have determined thousands of genes respond to BRs in *Arabidopsis*^54–56^. In this study, 2,147 upregulated and 768 downregulated genes in soybean under Pcz-BL-1h treatment shared homology with 1,345 and 554 *Arabidopsis* genes, respectively. In addition, 1,859 upregulated and 2,122 downregulated genes in soybean under Pcz-BL-8h treatment shared homology with 1,174 and 1,501 *Arabidopsis* genes, respectively. To assess the genetic diversity between the treatments within *Arabidopsis* and soybean, the responsive genes identified under each condition were compared. The results of this analysis are shown in Venn diagrams in Fig. S6. A total of 201 (20.0%) and 123 (13.3%) differentially response genes in *Arabidopsis* were identified to coincide with those in soybean, respectively. Furthermore, the analyses of these overlapping genes between *Arabidopsis* and soybean revealed a wide range of cellular transport-related, cell wall-related enzyme-encoding, transcription factor-encoding, metabolism-related, stress-related, and hormone-related genes. These results revealed that some developmental processes are conserved in the BR-response networks of soybean and *Arabidopsis*.

## Discussion

Here, we evaluated the soybean physiological response to BRs synthesis inhibitor and then performed a series of RNA-seq experiments to study the transcriptional response to BRs synthesis inhibitor. Analysis of the differentially expressed genes provides a fair amount of insight into understanding the changes in the soybean physiological response.

### BRs regulate a wide range of physiological and developmental processes in soybean

Mutations in *Arabidopsis* BRs biosynthesis caused de-etiolation phenotypes in the dark, indicating that BRs are essential regulators of photomorphogenesis. Similarly, Pcz treatment produced dark-green cotyledons in *Arabidopsis*^25^. Moreover, cell elongation was significantly inhibited in BR biosynthesis mutants^57,58^. In this study, Pcz treatment produced typical BR-deficient phenotypes of soybean seedlings, such as shortened roots, shoots, and petioles. In addition, the chlorophyll content significantly increased under Pcz treatment, but there was no significant difference under Pcz-BL treatment compared with the control. Furthermore, both transcriptional and physiological data correlated well with the findings in this research. For example, some PS-related genes were repressed by Pcz treatment, and the repression is reversed by BR applications. Pcz-mediated suppression of growth is likely the result of specific inhibition of the cell wall-related pathway. These results not only show that BRs play a conserved function in photomorphogenesis in both *Arabidopsis* and soybean but also provide evidence linking phenotype with gene expression levels.

However, this study focused on the response of soybean to BRs only at the early growth stage. In rice, BRs have been implicated in the stimulation of the source-to-sink flow of assimilates, leading to improved grain filling and larger seeds^59,60^. According to this study, BRs also had a significant impact on seed development. Therefore, future studies should focus on the regulation of gene expression during the reproductive growth stage.

### Quick-response genes were identified by comparing various treatments

Although BR-responsive genes have been extensively identified in *Arabidopsis* by several microarray studies^61–66^, little is known about the quick response of BRs. Identification of BR quick-responsive genes in soybean is vital for understanding the regulatory mechanism of gene expression. In this study, genes that exhibited opposite expression patterns between Pcz and Pcz-BL-1h or Pcz-BL-8h were extracted and analyzed (Tables S1 and S2). The results showed that these BR early-response genes contribute to amino acid metabolism, hormone metabolism, protein posttranslational modification and degradation, transporter, and cell wall pathways. These results support the fast regulation of BRs in soybean that allows prompt growth-related gene reprogramming and the establishment of an acclimation program, leading to specific morphophysiological responses and growth recovery. In addition, those reversely regulated genes between Pcz and Pcz-BL-1h may play as potential biomarkers for early detection of BR homeostasis changes. For example, *IAA19* is a target of BZR1 and induced by brassinolide in *Arabidopsis*^67,68^. The expression level of *IAA19* in soybean was reduced under Pcz and Pcz-BL treatments but highly induced under Pcz-BL-1h and Pcz-BL-8h treatments.

Genetics studies have revealed the roles of BZR1 and BZR2/BES1 in the BR synthesis feedback loop in *Arabidopsis*^32,54,55,69^. In a recent study, targets of the GmBZL3 transcription factor in soybean were identified via the ChIP-seq method^20^. Overlapping analysis between targets of GmBZL3 and DEGs induced under different BR levels indicated that the expression of GmBZL3 targets was strictly regulated by different BR levels in soybean. In the future, sequence enrichment analysis will be carried out to identify specific cis-elements in soybean via comprehensive analysis including targets of GmBZL3 and DEGs, which will help to elucidate the regulatory networks of BRs.

### Hormone interactions during the dynamic balance of BRs play important roles in regulatory networks under stress conditions

BRs interact with other hormones in controlling a wide range of abiotic stresses in plants, such as high temperature, salinity, and drought stress^50,70,71^. For example, overexpression of one of the BR biosynthesis genes, *DWF4*, in *Brassica napus* can simultaneously increase seed yield and plant tolerance to stress^72^. Moreover, CK levels and drought tolerance were enhanced in rice plants overexpressing the isopentyl transferase (*IPT*) gene, which corresponded with the upregulation of various BR-related biosynthesis genes (*DWF4*, *DWF5*, and *HYD1*) and BR signaling genes (*BRI1*, *BZR1*, *BAK1*, *SERK1*, and *BRH1*)^73^. Previous studies have shown that 2-oxoglutarate Fe(II)-dependent oxygenase superfamily proteins are involved in the ethylene metabolism process and plant defense response to drought^74^. Other studies have shown that hormone crosstalk plays an important role via interactions with abiotic stress-related pathways in soybean^53,75^. In this study, many genes encoding 2-oxoglutarate Fe(II)-dependent oxygenase and isopentyl transferase were regulated under various BR concentrations or water-deficit conditions. EDS1 and PAD4 proteins constitute a regulatory hub for gene-mediated and basal resistance and are required for the accumulation of SA in *Arabidopsis*^42,76,77^. The RNA level of *PAD4* increased under MS and SS conditions and was upregulated under Pcz-BL-8h treatment in this study. *EDS1* was downregulated under MS condition and upregulated under Pcz-BL, Pcz-BL-1h, and Pcz-BL-8h treatments (Table S4). These results indicated that hormone interactions and stress responses are involved in complex regulatory mechanisms. Therefore, the investigation of hormone interactions with BRs under water-deficit conditions in soybean will help elucidate drought regulatory networks. Further investigation is required to understand the mechanism of BR and other hormone crosstalk involved in drought stress tolerance in soybean. An in-depth understanding of the mechanism could stabilize soybean yields under changing climatic conditions.

## Methods

### Plant growth and RNA sample collection

The soybean genotype Williams 82 (Wm 82) was used for phenotypic analyses and RNA-seq experiments. Plants were grown in 26.5 L pots filled with a Turface and sand mixture (2:1). Osmocote (Scotts Co., Marysville, OH), with an N:P_2_O_5_:K_2_O fertilizer analysis of 14:14:14, was added as a nutrient source at a rate of 20 g per pot. The soybean plants were grown in a greenhouse under a 16-h photoperiod at 28 °C in well-watered conditions until the vegetative 1 (V1) stage. Tissue samples were collected from the following treatment conditions: V1-stage plants irrigated with water (Mock, 10 days), V1-stage plants irrigated with water containing a high concentration of BR synthesis inhibitor (5 μM propiconazole for 10 days, indicated as Pcz, Banner Maxx-60207-90-1, Syngenta, Greensboro, NC), or V1-stage plants subjected to a combined treatment of a high concentration of the inhibitor and a low concentration of BL (5 μM Pcz with 10 nM BL for 10 days, indicated as Pcz-BL). High-concentration BL was applied for 1 h or 8 h after 10 days of treatment with the high-concentration inhibitor (5 μM Pcz for 10 days followed by 1 μM BL for 1 h, indicated as Pcz-BL-1h; 5 μM Pcz for 10 days followed by 1 μM BL for 8 h, indicated as Pcz-BL-8h). Plant height, primary root length, total root length, leaf area, shoot length, and petiole length were measured after each treatment. The leaf chlorophyll content was measured by a chlorophyll meter (SPAD-502 Plus, Spectrum Technologies, Aurora, IL). Tissue samples and data were collected from three biological replicates.

### RNA isolation and library construction

Briefly, total RNA was isolated from the soybean seedlings with an RNeasy Plant Mini Kit (Qiagen, Cat#:74904), and on-column DNase digestion with an RNase-Free DNase kit was used to remove the DNA contamination (Qiagen, Valencia, CA, Cat#:79254). The RNA integrity was analyzed using an Agilent 2100 Bioanalyzer (RNA Nano Chip, Agilent, Santa Clara, CA). Only RNA with an integrity value greater than 8 was selected for library construction. An RNA TruSeq Stranded mRNA LT sample prep kit (Illumina, San Diego, CA, RS-122-2101) was used to prepare the RNA-seq library following the manufacturer’s protocol.

### RNA sequencing and data processing

Single-end reads were generated by the Illumina HiSeq 2000 platform (read length 1 × 100 bases; Illumina, Inc. San Diego, CA). The initial base calling and quality filtering of the reads generated with the Illumina analysis pipeline (Fastq format) were performed using a custom Perl script and the default parameters of the Illumina pipeline (http://www.illumina.com). Additional filtering for poor-quality bases was performed using the FASTX-toolkit available in the FastQC software package (http://www.bioinformatics.babraham.ac.uk/projects/fastqc/). Only uniquely mapped reads were used in the analysis. Two mismatched base pairs were allowed, and the multiple position matching was reported up to 40 alignments using the TopHat mapping procedure. High-quality mRNA-seq reads were then aligned to the *Glycine max* reference genome (Gmax1.1 version) and Phytozome v9.0 gene model release using the TopHat software package with the default parameters (version 140 1.4.1)^78^. The genome indexes for TopHat were constructed using the Bowtie “build” command of Bowtie (version 0.12.7), with the reference genome file used as an input^79^.

### Sequence data analysis and differential counting

Sequence data analysis and differential counting were conducted as described previously^53,75^. The gene expression (FPKM) levels were estimated using Cufflinks software (version 2.1.1)^80^, while differential gene expression analysis was performed using Cuffdiff (version 2.1.1) among the different sample comparisons. Only the genes with a log2-fold change ≥+2 and ≤−2 but without infinite values and an FDR adjusted *P* value of ≤5e-5 after Benjamini-Hochberg correction for multiple testing with a significance level of “yes” were considered significantly DEGs. The transcriptome raw sequencing data and analyzed gene expression results are available in the NCBI Sequence Read Archive via Bioproject accession PRJNA525277. The different levels of BR-response pathways were identified and plotted by MapMan^33^. In addition, we generated a heat-map using Multi Experiment Viewer (MeV) soft-ware (Version 4.9.0) and a hierarchical clustering (HCL) method for gene sorting.

### Expression profiling using RNA-seq datasets

The expression profile information of hormones and biotic/abiotic stress pathways in this study was extracted from previous data generated by Song *et al*. (2016) for Williams 82 plants subjected to VMS, MS, SS and SR conditions. Briefly, Williams 82 plants were kept well-watered until the V3 (three unfolded trifoliate leaves) stage. For VMS, drought stress was imposed by withholding water for 5 days. For MS, drought stress was imposed by withholding water for 12 days. For SS, drought stress was imposed by withholding water for 19 days. Last, for SR, the plants were re-watered for 2 days after withholding water for 19 days. Each treatment had corresponding control plants, and all control plants were kept well-watered until sampling.

### Quantitative real-time PCR assays

Total RNA was isolated from soybean seedlings with an RNeasy Plant Mini Kit (Qiagen, Valencia, CA, Cat#:74904). On-column DNase digestion with an RNase-Free DNase kit was used to remove any contaminated DNA (Qiagen, Valencia, CA, Cat#:79254). A High-Capacity cDNA Reverse Transcription Kit (Thermo, Waltham, MA, Cat#4368814) was used for cDNA synthesis. The qRT-PCR assay was carried out using SYBR Green Master Mix (Thermo, Waltham, MA, Cat# K0223). The comparative Ct method was used to quantify the relative expression of specific genes^81^. The cyclin gene (Glyma10g263500) was selected as an internal control to normalize gene expression. The relative expression measurements from the qRT-PCR were transformed into fold changes by base-2 conversion to match the RNA-seq fold change value. All primers used were designed using the Primer3 web interface (http://frodo.wi.mit.edu/primer3/input.htm; ^82^). The primer sequences used are listed in Supplementary Table 5. The reactions were performed with three biological replicates and were repeated once as a technical replicate.

## Supplementary information

Supplementary Figures

Supplementary table 1

Supplementary table 2

Supplementary table 3

Supplementary table 4

Supplementary table 5

## Data Availability

The data sets supporting the results of this article are included within the article (and its Supplementary Files). Illumina sequences data and processed RNA sequences data were deposited to the NCBI Sequence Read Archive through Bioproject accession PRJNA525277 (https://www.ncbi.nlm.nih.gov/bioproject/PRJNA525277).
