# Supplementary material for: Genome-wide transcriptional profiling for elucidating the effects of brassinosteroids on Glycine max during early vegetative development
Source: Sci Rep. 2019 Nov 6;9:16085. doi: 10.1038/s41598-019-52599-3 (PMC6834599; doi:10.1038/s41598-019-52599-3)
Supplement: Supplementary file 1 — Supplementary Figures [file 41598_2019_52599_MOESM1_ESM.pdf]

Supplementary Information

Genome-wide transcriptional profiling for elucidating the effects of brassinosteroids on *Glycine max* during early vegetative development

Li Song[1#*], Wei Chen[2#], Qiuming Yao[3], Binhui Guo[1], Babu Valliyodan[2], Zhiyong Wang[4], Henry T. Nguyen[2*]

[1]Joint International Research Laboratory of Agriculture and Agri-Product Safety, Jiangsu Key Laboratory of Crop Genomics and Molecular Breeding, Yangzhou University, Yangzhou, 225009, China

[2]Division of Plant Sciences, University of Missouri, Columbia, MO 65211, USA

[3]Department of Computer Science, Christopher S. Bond Life Sciences Center, University of Missouri, Columbia, MO 65211, USA

[4]Department of Plant Biology, Carnegie Institution for Science, Stanford, CA 94305, USA

[#] Equal contribution

Correspondence:

Dr. Li Song, Email: songli@yzu.edu.cn;

Dr. Henry T. Nguyen, Email: nguyenhenry@missouri.edu

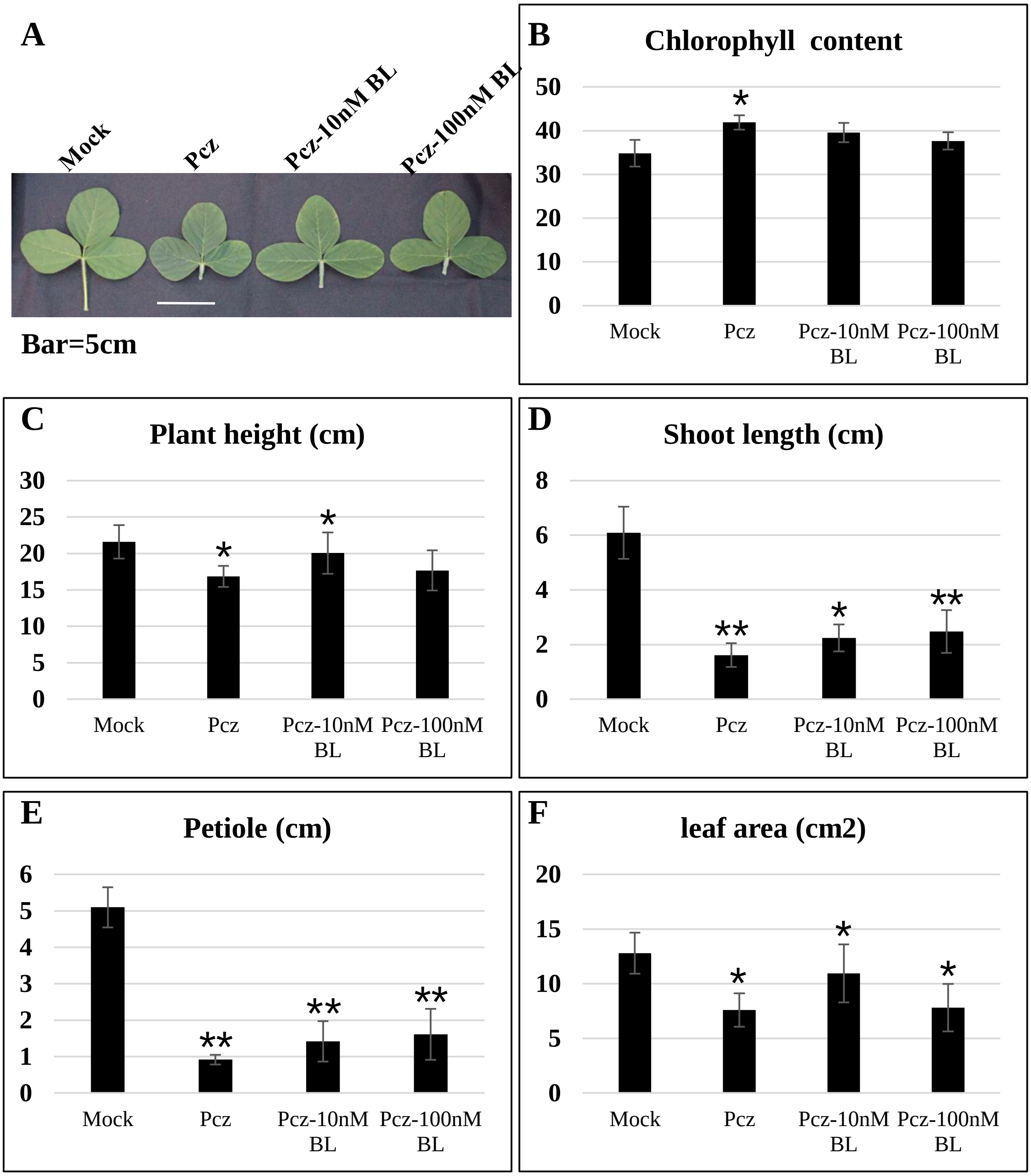

Supplementary Figure S1. **Responses of soybean seedlings to inhibitor treatments and BL complementation.** (**A**) Wm82 grows to V1 stage and then irrigated with 5µM Pcz or 5µM Pcz with 10nM BL or 100nM BL for 10 days. (**B-F**) Chlorophyll content, plant height, shoot length, petiole and leaf area were monitored after treatments. (n≥30). Statistical differences are marked with * ($p \leq 0.05$) or ** ($p \leq 0.01$) based on Student's *t*-test analysis.

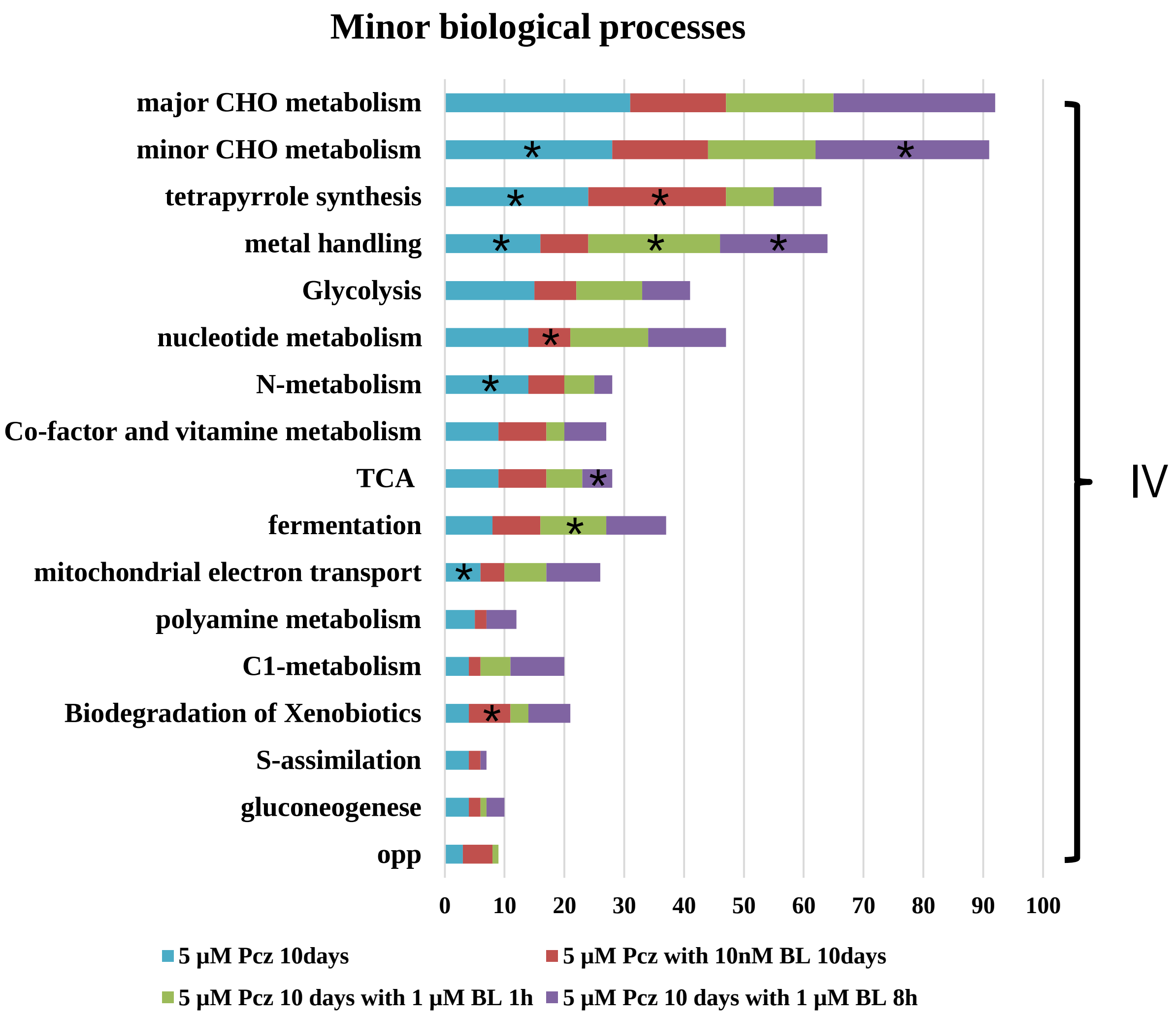

Supplementary Figure S2. GO term assignment to the transcripts in different categories (group IV) of minor biological process. Different color indicates number of genes involved in different conditions. Group IV<100. Statistical significance of each process under all treatments were performed by Fisher's exact test. The overrepresented categories of biological process (P values ≤0.05) were labeled with star (*).

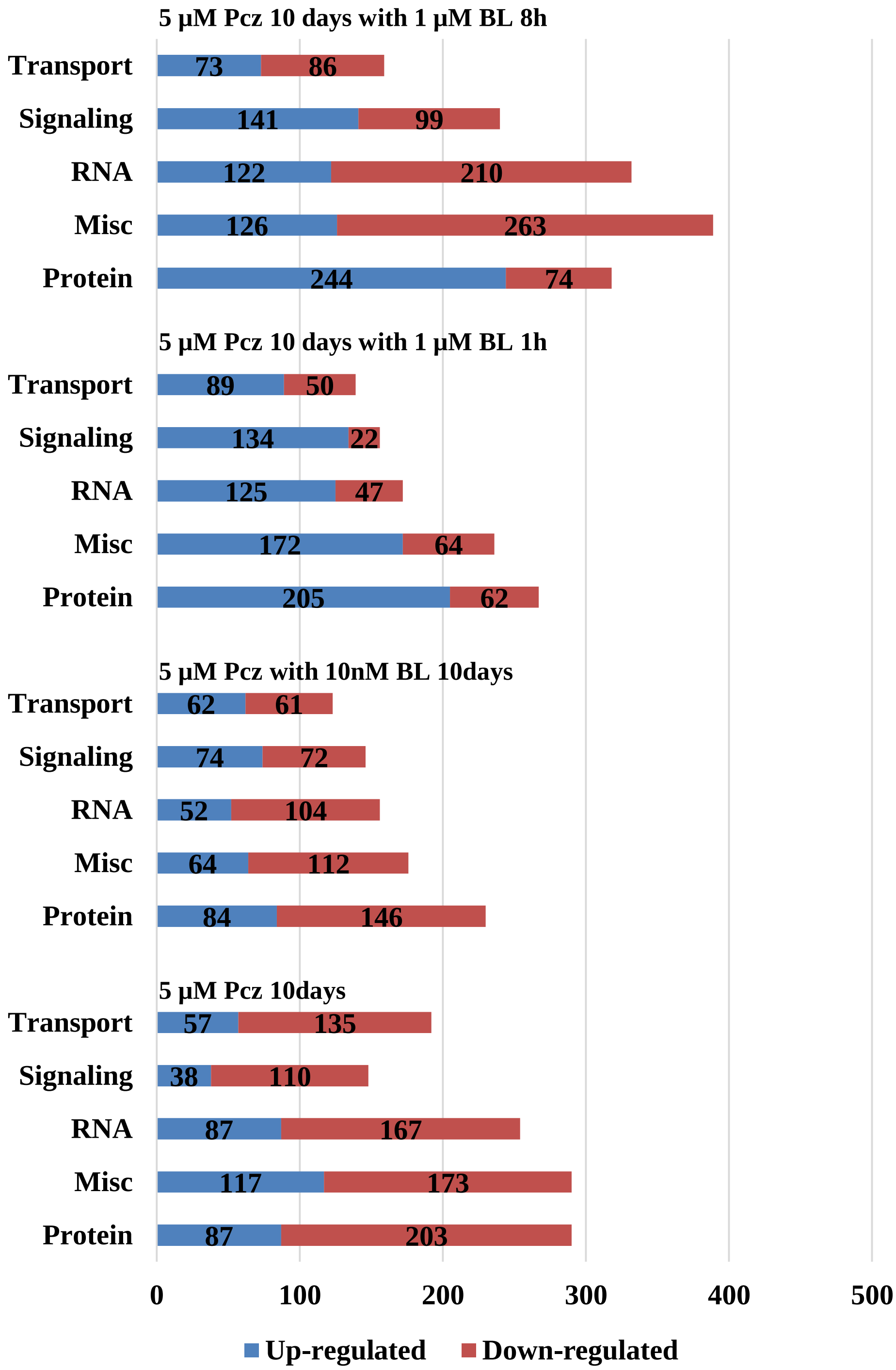

Supplementary Figure S3. Response patterns of functional categories (transport, signaling, RNA, miscellaneous and protein) under different treatments. Within each bin, the proportions of genes up- or downregulated are coded blue and red, respectively.

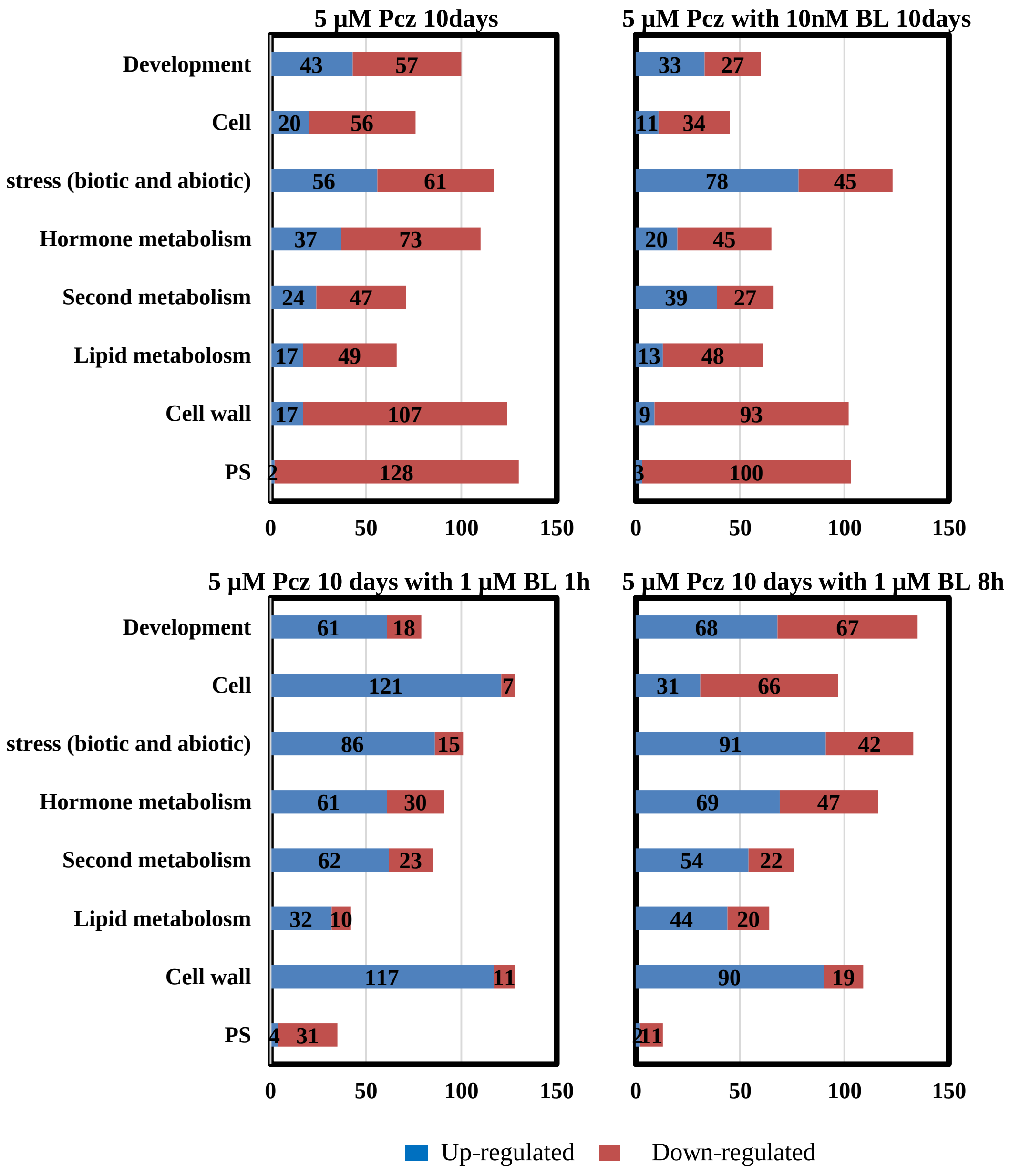

Supplementary Figure S4. Functional categories (development, cell, stress, hormone metabolism, second metabolism, lipid metabolism, cell wall and PS) responding differently treatments. Within each bin, the proportions of genes up- or down-regulated are coded blue and red respectively.

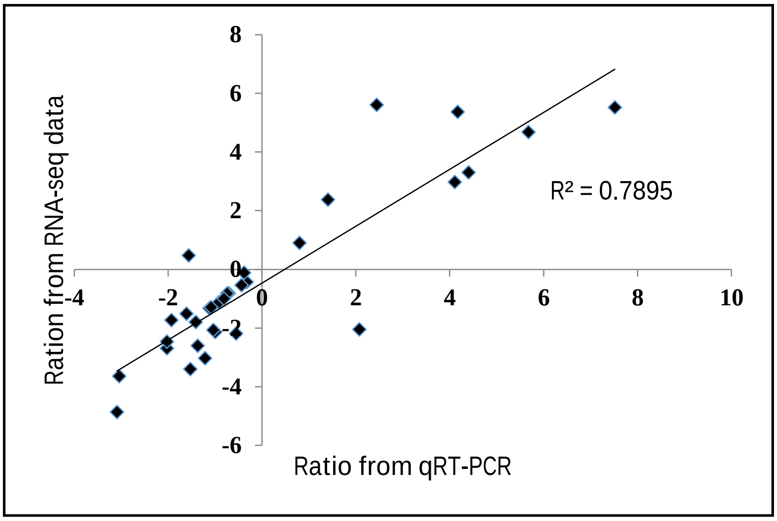

Supplementary Figure S5. Coefficient analysis indicated RNA-Seq and qRT-PCR results had a high correlation using the log2 values for different BRs levels. Each dot represents the average values for each gene from all the biological replicates.

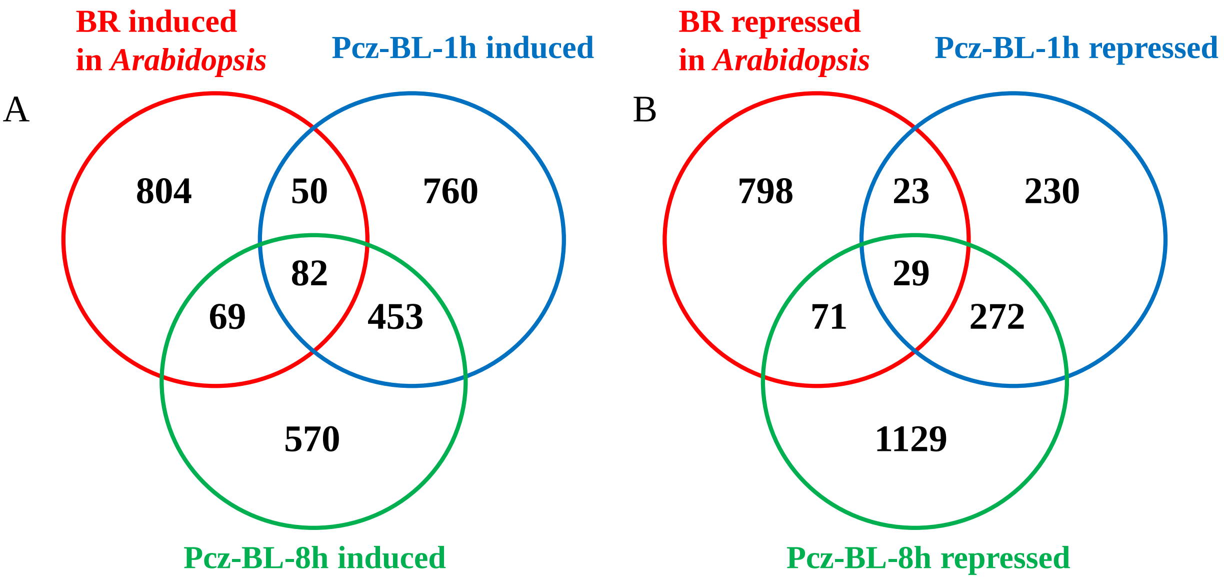

Supplementary Figure S6. Venn diagram showing the overlap of BR induced or repressed genes in *Arabidopsis* and soybean. (**A**) Comparison of BR induced genes in *Arabidopsis* and Pcz-BR-1h or Pcz-BR-8h treated soybean. (**B**) Comparison of BR repressed genes in *Arabidopsis* and Pcz-BR-1h or Pcz-BR-8h treated soybean. The response gene list comes from Sun et al., (2010).
